# Prenatal mTOR-Inhibitor Therapy for Fetal Cardiac Rhabdomyomas: Indications, Treatment Duration and Perinatal Outcomes

**DOI:** 10.3390/jcm15166453

**Published:** 2026-08-20

**Authors:** Ioannis Kyvernitakis, Katharina Schramm, Gert Wiegand, Bernd Feyerabend, Ahmet Alexander Baschat

**Affiliations:** 1 Section for Prenatal Diagnosis and Fetal Therapy, Asklepios Klinik Barmbek, Asklepios Klinik Nord-Heidberg, Barmbek Ruebenkamp 220, 22307 Hamburg, Germany; schrammkatharina81@gmail.com; 2 Section for Pediatric Neurology, Asklepios Klinik Nord-Heidberg, 22417 Hamburg, Germany; g.wiegand@asklepios.com; 3 Hansehistologikum Hamburg, 22547 Hamburg, Germany; feyerabend@pathologie-hh.de; 4 Center for Fetal Therapy, Johns-Hopkins University, Baltimore, MD 21287, USA; abascha1@jhmi.edu

**Keywords:** fetal cardiac rhabdomyoma, tuberous sclerosis complex, sirolimus, everolimus, mTOR inhibitor, fetal therapy, outflow tract obstruction, hydrops fetalis

## Abstract

**Background:** Fetal cardiac rhabdomyomas are strongly associated with tuberous sclerosis complex (TSC). Although many remain asymptomatic and regress spontaneously, large tumors may cause ventricular inflow or outflow obstruction, arrhythmia, impaired ventricular function, pericardial effusion, hydrops fetalis and fetal demise. Transplacental mammalian target of rapamycin (mTOR) inhibition has emerged as a potential rescue therapy. **Methods:** We performed a narrative review of published reports on prenatal sirolimus or everolimus therapy for fetal cardiac rhabdomyomas in suspected or confirmed TSC. Data was extracted on treatment indication, gestational age at initiation, treatment duration, fetal echocardiographic response, maternal adverse effects, delivery and postnatal outcome. **Results:** Available evidence consists predominantly of case reports, small case series and retrospective cohorts; no prospective controlled trials were identified. Treatment was generally initiated for progressive or hemodynamically significant disease, particularly ventricular inflow or outflow obstruction, worsening valve regurgitation, arrhythmia, pericardial effusion, ventricular dysfunction or hydrops. Therapy was usually started in the late second or third trimester and continued until hemodynamic stabilization, delivery or planned transition to neonatal treatment. Most reports described tumor regression within 1–3 weeks, accompanied by improved cardiac function and high perinatal survival. However, rebound growth after treatment withdrawal, persistent arrhythmic risk and limited long-term safety data remain important concerns. **Conclusions:** Prenatal mTOR-inhibitor therapy should be considered an individualized rescue or stabilization strategy for fetuses with life-threatening or progressive cardiac compromise, rather than routine treatment for all fetal rhabdomyomas. Management should be multidisciplinary and guided by fetal hemodynamics, treatment response, maternal tolerance and gestational age.

## 1. Introduction

**Figure 1:**
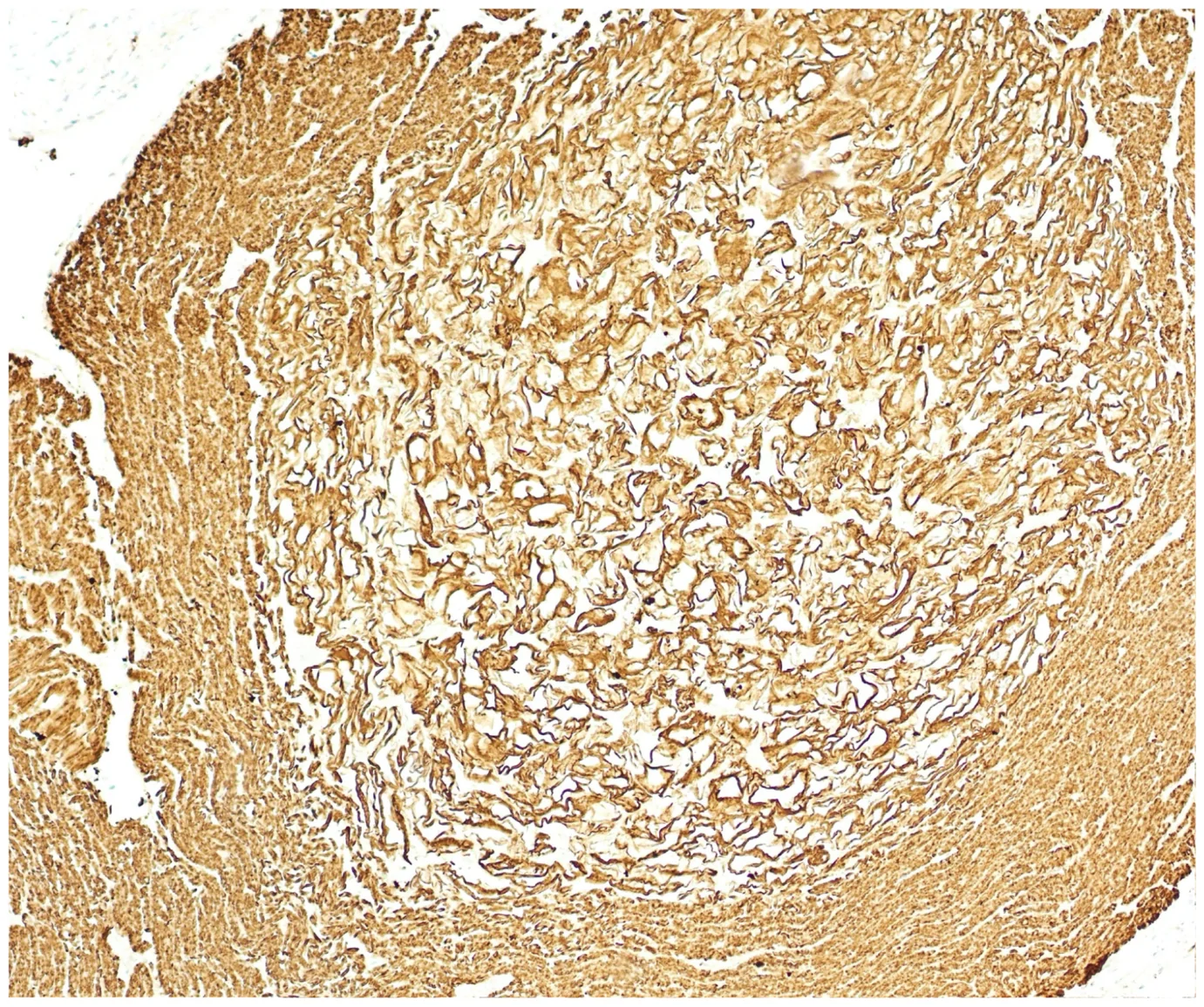
Cardiac rhabdomyoma showing a distinct architectural transition from the surrounding myocardium, highlighted by desmin immunohistochemistry. Original magnification, ×200.

Cardiac rhabdomyomas are the predominant primary cardiac tumors of fetal life. Although histologically benign (Figure 1), their clinical relevance is determined by location, growth trajectory and interaction with intracardiac flow rather than by histology. In contemporary series, most fetuses remain stable, but outflow tract obstruction, arrhythmia, pericardial effusion, hydrops and fetal death occur in a clinically relevant minority [1,2]. Multiple lesions are highly suggestive of TSC, and a cardiac rhabdomyoma itself represents a major diagnostic feature of the disorder [3].

The conventional prenatal approach has been surveillance, because spontaneous stabilization in late gestation and postnatal regression is common. This remains appropriate for most fetuses. The management problem arises when a tumor occupies a critical position within the ventricular cavity, enlarges rapidly before fetal maturity or is associated with declining cardiac performance. In such cases, expectant management may culminate in hydrops, urgent preterm delivery or a neonate with critical obstruction that cannot be managed by maintaining ductal patency alone.

Transplacental mTOR inhibition offers a biologically targeted alternative. Since the first report of maternal sirolimus in 2018, prenatal therapy has been described in a limited but steadily expanding number of fetuses [4,5,6,7,8,9,10,11,12,13,14,15,16,17,18,19,20]. The striking regression reported in many cases has generated considerable enthusiasm; however, the available literature remains vulnerable to selection and publication bias, and treatment protocols differ substantially. The clinically relevant questions are therefore not whether a rhabdomyoma can respond to an mTOR inhibitor, but which fetus requires treatment, when therapy should begin, how long it should be continued and what outcome can reasonably be expected.

## 2. Literature Identification Search Strategy and Study Selection

For this narrative review, PubMed/MEDLINE and the reference lists of relevant reviews were searched for reports published from the first prenatal treatment report in 2018 to 1 June 2026. Search terms included combinations of “fetal”, “prenatal”, “cardiac rhabdomyoma”, “tuberous sclerosis”, “sirolimus”, “everolimus” and “mTOR inhibitor”. Case reports, case series, systematic or narrative reviews and cohorts were considered when prenatal maternal exposure and fetal or neonatal outcome were described. Reports of exclusive postnatal treatment were not included in the primary evidence table. The search was conducted in line with the PRISMA guidelines. The search term “tuberous sclerosis” resulted in 32,287 publications. The search term “Tuberous sclerosis + prenatal therapy” resulted in 2561 publications. When defining “tuberous sclerosis +prenatal therapy+ humans”, 2375 results were found. When limiting this to “TSC prenatal therapy”, only 56 studies were identified. This was the database we further worked on. We only included English publications since this is the language that is internationally most accepted. Duplicates were extracted.

The published evidence remains non-randomized. A 2024 systematic review identified 11 prenatally treated fetuses in nine reports [2]. Subsequent reviews expanded the experience to 15 fetuses and then to 21 exposed fetuses, including two small series and a twin pregnancy [20,21]. In 2026, a single-center retrospective cohort of 27 pregnancies, 7 of which received prenatal sirolimus, provided the first comparative longitudinal data on tumor growth and treatment duration [22].

## 3. Risk Stratification

The natural course is central to treatment selection. Many fetal rhabdomyomas enlarge during the second and early third trimesters, then stabilize or regress after birth. In the 2026 cohort, untreated tumors grew most rapidly between 20 + 0 and 27 + 6 weeks, whereas growth slowed after 28 weeks [22]. Consequently, an isolated measurement is less informative than serial assessment of growth, intracardiac position and functional effect.

Before maternal immunosuppressive therapy is initiated, the diagnosis of cardiac rhabdomyoma should be as reliable as possible. Differential diagnosis may include cardiac fibroma, teratoma or other rare cardiac masses. When a genetic diagnosis via amniocentesis confirms TSC1 or TSC2, the proposed treatment algorithm can be initiated [21].

Tumor size remains relevant but is not a sufficient treatment criterion. A relatively modest septal lesion may compromise both outflow tracts or create an arrhythmogenic substrate, whereas a larger intramyocardial lesion may have little hemodynamic effect. The decision to intervene should therefore integrate: (i) the direction and velocity of growth; (ii) proximity to the atrioventricular valves and ventricular inflow/outflow tracts; (iii) antegrade flow and Doppler evidence of obstruction; (iv) ventricular filling and systolic performance; (v) atrioventricular valve regurgitation; (vi) rhythm; and (vii) signs of venous congestion, pericardial effusion or hydrops.

**Figure 2:**
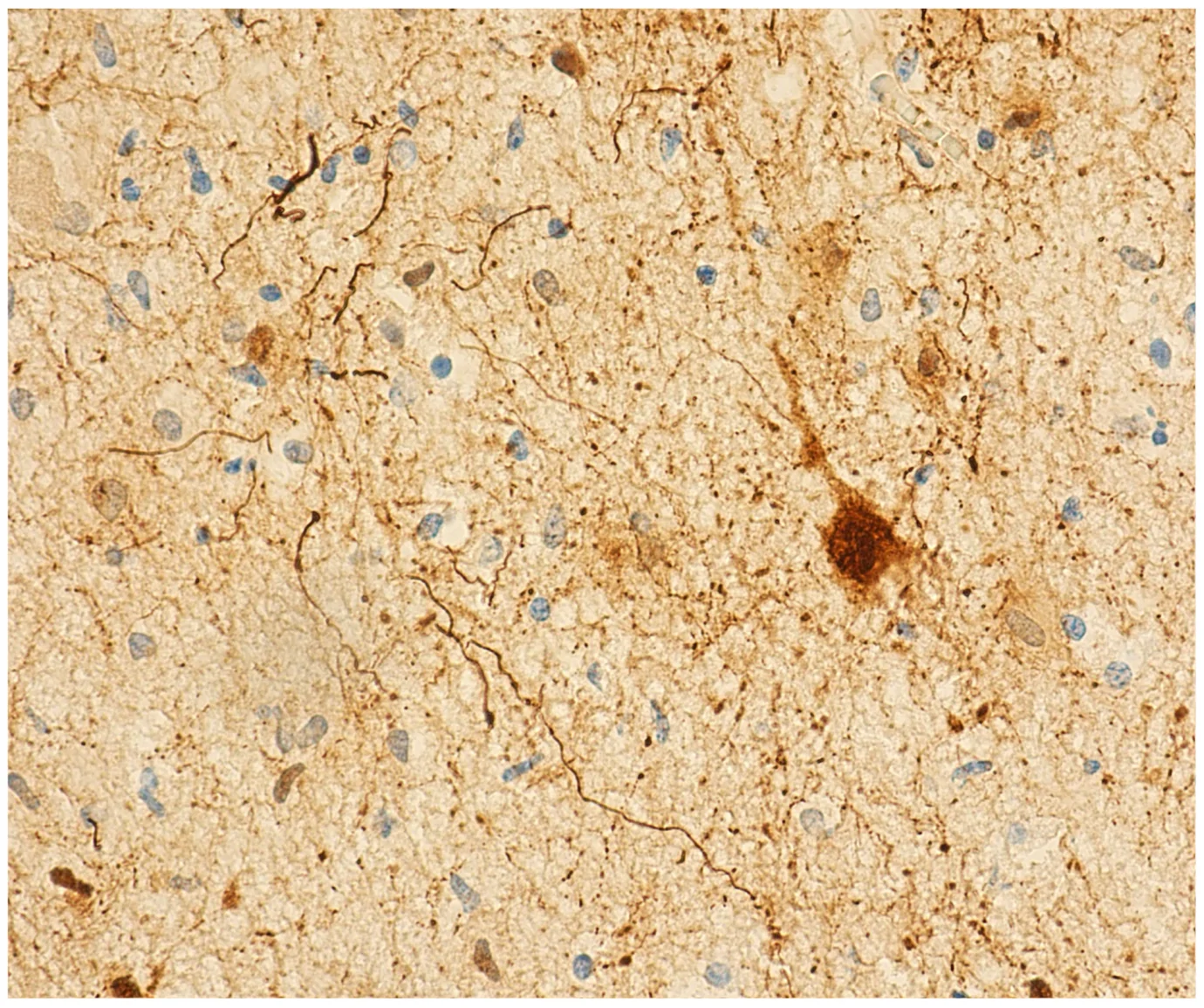
Giant astrocyte highlighted by immunohistochemical staining for glial fibrillary acidic protein (GFAP). Original magnification, ×400.

Expectant management remains the default when cardiac output is preserved, no critical structure is compromised, and serial examinations show stability. In such cases, maternal exposure to an off-label immunosuppressive agent cannot be justified solely by the presence of TSC, multiple tumors or parental concern regarding future neurological disease (Figure 2).

## 4. Biological Rationale for Transplacental mTOR Inhibition

TSC results from pathogenic variants in TSC1 or TSC2, leading to constitutive activation of the mTOR pathway and dysregulated cellular growth. Sirolimus and everolimus inhibit this pathway and reduce the volume of several TSC-associated lesions. Prenatal treatment extends an established postnatal principle to the fetus through maternal administration and placental transfer. Measurable drug concentrations in cord blood confirm fetal exposure [4,7,12,15].

The intended prenatal endpoint is not necessarily complete disappearance of the tumor. In a compromised fetus, a moderate reduction in mass may be sufficient to restore antegrade flow, improve ventricular filling, reduce atrioventricular valve regurgitation and interrupt the progression from low cardiac output to hydrops. Fetal therapy is therefore best understood as hemodynamic rescue or a bridge to a safer gestational age, rather than definitive treatment of TSC.

## 5. Indications for Fetal Therapy

### 5.1. A Physiology-Based Rather than Size-Based Indication

Published treatment decisions have generally been prompted by progressive tumor growth combined with actual or impending cardiovascular compromise. The threshold should be highest when the fetus is near term and delivery followed by neonatal treatment is a reasonable alternative, and lower when deterioration occurs at a gestational age at which delivery would impose substantial prematurity-related morbidity. A multidisciplinary decision should explicitly compare three competing strategies: continued surveillance, transplacental treatment and delivery.

### 5.2. Ventricular Outflow Tract Obstruction

Left- or right-ventricular outflow tract obstruction is the most consistent indication in the literature [4,7,8,11,14,16,17,18,19,20]. A tumor protruding into the outflow tract may progressively reduce antegrade flow, increase ventricular pressure, impair systolic performance and produce secondary atrioventricular valve regurgitation. Bilateral involvement is particularly concerning because maintenance of ductal patency after birth cannot compensate reliably for simultaneous compromise of both ventricular outputs.

Treatment should be considered when obstruction is established or clearly evolving, especially when accompanied by increasing Doppler velocity, reduced forward flow, ventricular dysfunction, cardiomegaly, valve regurgitation or abnormal venous Doppler. The aim is to intervene before persistent low output results in hydrops or before urgent delivery becomes unavoidable. The 2026 cohort supports the need for sustained exposure: treatment for more than 7 days was associated with tumor regression and/or resolution of outflow obstruction, whereas shorter exposure was not [22].

### 5.3. Ventricular Inflow Obstruction

**Figure 3:**
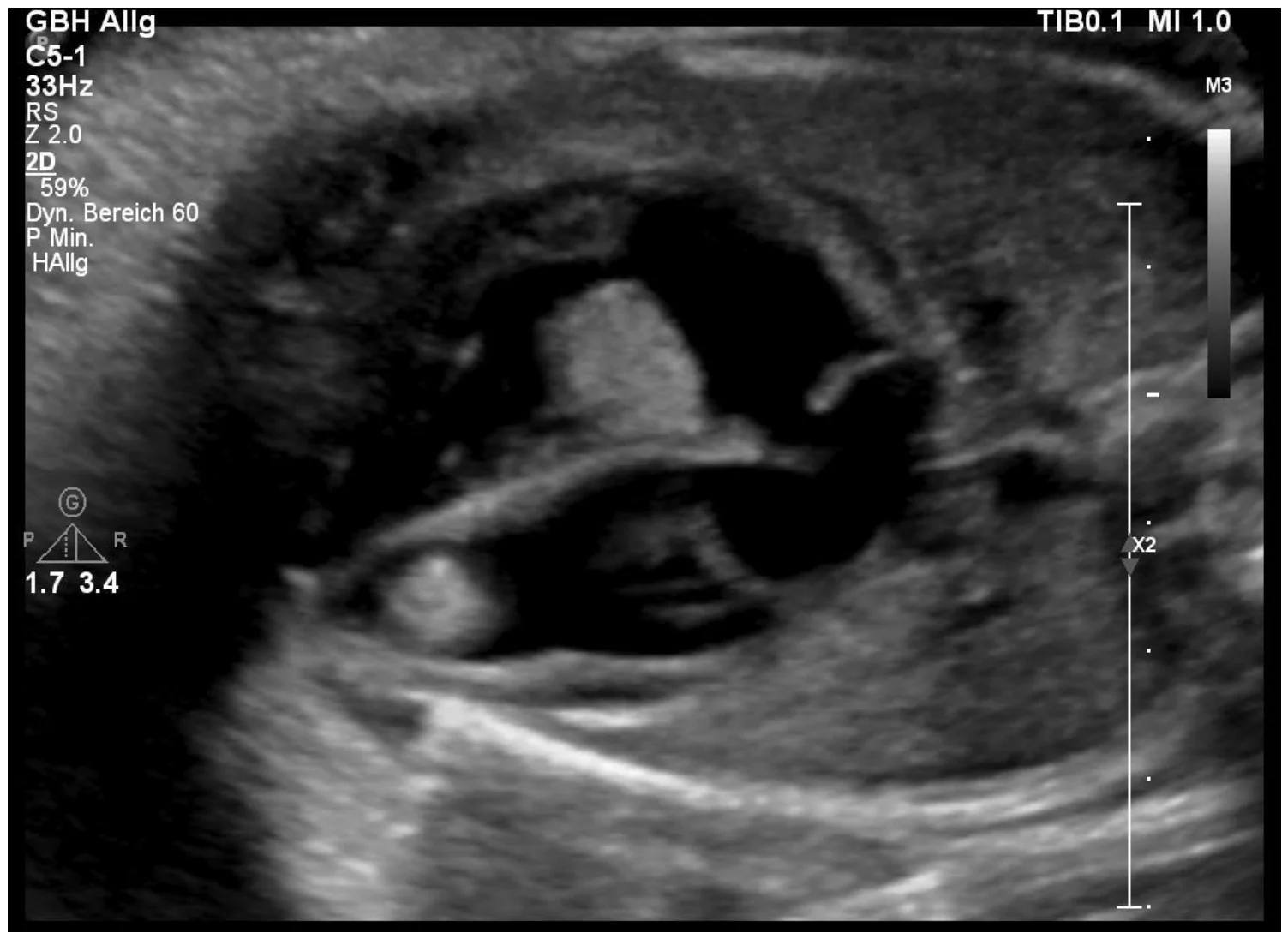
Fetal echocardiography demonstrates two large cardiac rhabdomyomas causing partial obstruction of the ventricular outflow tracts.

Inflow compromise receives less attention than outflow obstruction but may be equally important (Figure 3). Tumors adjacent to the tricuspid or mitral valve can restrict diastolic filling, distort the annulus or generate severe regurgitation. Right-sided inflow obstruction increases systemic venous pressure; left-sided obstruction reduces systemic preload and output. The report by Will et al. illustrates that reduction of a tumor partially covering the tricuspid valve may restore effective filling and permit term delivery [12].

Clinically relevant inflow obstruction, particularly when associated with atrioventricular valve regurgitation, ventricular dysfunction, abnormal ductus venosus flow or developing hydrops, represents a strong indication for treatment. Mere contact between a tumor and a valve, in the absence of functional impairment, does not.

### 5.4. Hydrops Fetalis and Evolving Cardiac Failure

Hydrops is the clearest rescue indication. It reflects advanced failure of the fetal circulation and may result from obstruction, impaired contractility, sustained tachyarrhythmia or a combination of these mechanisms. Reversal of hydrops after maternal sirolimus has been reported in singleton and twin pregnancies [11,18]. In the retrospective cohort, hydrops occurred in association with large tumors and rapid growth, underscoring that both absolute burden and trajectory matter [22].

Waiting for fully developed hydrops is undesirable. Progressive pericardial effusion, worsening atrioventricular valve regurgitation, falling ventricular function, cardiomegaly, abnormal venous Doppler or a declining cardiovascular profile score should be interpreted as evidence of evolving failure. When these findings progress despite close surveillance, therapy may be justified before generalized hydrops develops [5,7,15,16,17].

### 5.5. Tumor-Associated Arrhythmia

Rhabdomyomas can provide an anatomical substrate for pre-excitation and re-entrant tachycardia. Arrhythmia has been an indication for prenatal therapy in several reports and may improve as tumor burden decreases [4,9,19]. Standard transplacental antiarrhythmic treatment remains appropriate when the rhythm disorder is isolated and controllable. mTOR inhibition becomes more compelling when arrhythmia coexists with obstruction, ventricular dysfunction or hydrops, or when recurrent tachycardia appears mechanistically linked to a large septal or atrioventricular-junction tumor.

Tumor regression should not be equated with elimination of arrhythmic risk. Muschel et al. reported a fetus in whom prenatal tumor progression and impending bilateral outflow obstruction were controlled during sirolimus exposure, but therapy was discontinued because of maternal respiratory symptoms. The neonate subsequently developed refractory re-entrant tachyarrhythmia, capillary leak and multiorgan failure and died on day 7 [20]. This case is a necessary counterweight to the predominantly favorable published experience and argues for explicit postnatal rhythm surveillance and a planned transition strategy.

### 5.6. Rapid Progression Before Fetal Maturity

Rapid growth alone is a conditional, not absolute, indication. It becomes clinically relevant when the tumor is approaching a critical inflow or outflow structure, when the gestational age makes delivery undesirable, or when early functional changes are already present. The expected natural deceleration of growth after 28 weeks may support continued surveillance in a stable fetus; conversely, a tumor growing several millimeters per week in the mid-second trimester may justify earlier treatment because the fetus has little reserve for further enlargement [22].

### 5.7. Situations in Which Treatment Is Generally Not Justified

Prenatal mTOR inhibition should not be offered routinely for an incidental, non-obstructive rhabdomyoma with preserved cardiac function. Neither multiple tumors nor a confirmed TSC1/TSC2 variant is, by itself, an indication. The report by Park et al., in which maternal sirolimus was required for maternal lymphangioleiomyomatosis and fetal tumors regressed, demonstrates biological efficacy but should not be interpreted as evidence for prophylactic treatment of an otherwise stable fetus [6]. Current evidence also does not support treatment solely to prevent cortical tubers, epilepsy or neurodevelopmental impairment.

## 6. Timing and Duration of Therapy

### 6.1. When to Start

In the published experience, treatment was initiated between approximately 23 and 35 weeks [4,5,6,7,8,9,10,11,12,13,14,15,16,17,18,19,20]. The appropriate timing is determined less by a fixed gestational-age threshold than by the balance between the rate of deterioration and the alternatives available. Treatment should begin early enough to permit a biological response; reduction or stabilization is often first evident after 1–3 weeks. Initiating treatment only when delivery is imminent may expose the mother without allowing sufficient time for fetal benefit.

Before approximately 34 weeks, fetal therapy may avoid extreme or very preterm delivery. Near term, delivery and direct neonatal therapy may be preferable when the fetus is stable, and immediate pediatric cardiology support is available. Hydrops, severe obstruction, or rapidly declining function should override an otherwise conservative gestational-age approach.

### 6.2. Treatment Duration

No standardized protocol has been established. Reported courses range from several weeks to treatment through delivery, with maternal target trough concentrations commonly between 5 and 15 ng/mL. The 2026 cohort suggests that courses of 7 days or less are unlikely to influence tumor size or established complications [22]. A more clinically useful principle is to continue treatment until the fetal objective has been achieved and a safe exit strategy has been defined.

For a fetus treated because of obstruction or hydrops, improvement alone should not automatically prompt withdrawal. Rebound growth has been described repeatedly after prenatal cessation and was observed in the cohort [4,5,14,21,22]. If a substantial residual tumor remains in a critical position, continued treatment until delivery is generally more defensible than early discontinuation. When maternal toxicity necessitates withdrawal, surveillance should be intensified and delivery or neonatal re-initiation planned proactively.

## 7. Dosing, Maternal Safety and Fetal Monitoring

Sirolimus has been used in most prenatal cases; everolimus has been reported less frequently [10,13]. Regimens have varied substantially in loading dose, maintenance dose and target concentration. Most centers have titrated maternal whole-blood trough concentrations to approximately 5–15 ng/mL, but a fetal response has also occurred below the intended target [20,23,24]. These observations do not establish an optimal therapeutic range. Maternal pharmacokinetics, placental transfer and fetal exposure are insufficiently characterized to support a single dosing protocol.

Baseline assessment should include complete blood count, renal and hepatic function, fasting lipid profile or triglycerides, glucose and blood pressure. During treatment, drug levels and laboratory surveillance should initially be performed at least weekly, with particular attention to cytopenia, transaminitis, proteinuria, dyslipidemia, hyperglycemia, infection, oral ulceration and respiratory symptoms. Maternal aphthous lesions, stomatitis, hypertriglyceridemia, gestational diabetes and possible pulmonary toxicity have been reported [12,14,15,16,20]. Fetal growth and amniotic fluid require surveillance because fetal growth restriction and oligohydramnios have occurred in individual pregnancies, although causality cannot be inferred from isolated reports [7,8,11].

Because both agents are used off-label in pregnancy and direct comparative data are lacking, sirolimus and everolimus should not be considered interchangeable. Potential interactions with CYP3A4 and P-glycoprotein inhibitors or inducers should be reviewed before and during therapy. Decisions on dose reduction or interruption around delivery should be individualized because evidence is limited; maternal infection, cytopenia, impaired wound healing, drug half-life, mode and urgency of delivery, and the fetal risk of withdrawal should all be considered.

Fetal safety assessment should include serial growth, amniotic-fluid volume and placental function in addition to cardiac surveillance. After birth, exposed neonates should undergo clinical examination, echocardiography and rhythm monitoring, with laboratory assessment—such as blood count, renal and hepatic function, glucose and lipids—guided by the extent and timing of exposure and the neonatal condition. Surveillance for infection is appropriate. Evidence is insufficient to define universal recommendations for breastfeeding or live vaccination after prenatal exposure; these decisions should be individualized with neonatology, pediatric infectious-disease/pharmacy expertise and current product information, particularly if maternal or neonatal mTOR-inhibitor therapy continues after delivery.

Fetal monitoring should include standardized echocardiographic assessment of, when appropriate:

- tumor number, location, and dimensions measured in reproducible imaging planes;
- tumor growth rate on serial examinations;
- degree of ventricular inflow or outflow tract obstruction;
- Doppler velocity and estimated gradient, with attention to the angle of insonation;
- severity of atrioventricular or semilunar valve regurgitation;
- qualitative and quantitative assessment of right and left ventricular function;
- cardiothoracic area ratio;
- Cardiovascular Profile Score;
- ductus venosus flow;
- umbilical venous pulsation;
- pericardial or pleural effusion;
- should document tumor dimensions in reproducible planes, the relationship to valves and inflow/outflow tracts, antegrade flow, peak velocities when interpretable, ventricular systolic function, atrioventricular valve regurgitation, rhythm, pericardial effusion and signs of hydrops. Ductus venosus and other venous Doppler measurements are useful when congestion is suspected. Weekly assessment is reasonable after treatment initiation; more frequent review may be required for hydrops, sustained arrhythmia or rapidly deteriorating function.

## 8. Fetal, Perinatal and Postnatal Outcomes

### 8.1. Fetal Response

Across the published cases, tumor stabilization or regression is the most consistent observation. Functional improvement has included relief of LVOT or RVOT obstruction, restoration of ventricular filling, improved ejection, reduction in atrioventricular valve regurgitation, resolution of pericardial effusion and reversal of hydrops [4,5,7,8,9,10,11,12,13,14,15,16,17,18,19,20]. The response often becomes apparent within 2 weeks, although complete regression is neither necessary nor expected.

The systematic review by Mustafa et al. found no fetal or neonatal deaths and no cardiac surgery among the 11 prenatally treated fetuses identified at that time, contrasting with adverse outcomes in the broader expectantly managed literature [2]. This comparison is hypothesis-generating rather than causal: treated cases were highly selected, the studies were uncontrolled and successful experiences were more likely to be published. The subsequent neonatal death reported by Muschel et al. demonstrates that the early literature did not capture the full risk spectrum [20].

### 8.2. Delivery and Neonatal Transition;

Most reported pregnancies reached the late-preterm or term period. Vaginal delivery is feasible when fetal status is stable, and there is no obstetric contraindication; cesarean delivery has often reflected concern regarding neonatal compromise or unrelated obstetric indications rather than the tumor itself. Delivery should occur in a center with neonatology, pediatric cardiology and access to intensive care. Immediate echocardiography should define residual obstruction, ventricular function and the need for postnatal treatment.

A planned prenatal-to-postnatal transition is particularly important when treatment is stopped before delivery or when a large residual lesion remains. Several infants received sirolimus or everolimus postnatally, either electively or after rebound growth [4,11,12,13,14,15,16,17]. Postnatal therapy should not be based solely on persistence of a mass; the same physiological criteria—obstruction, dysfunction, arrhythmia and clinical instability—remain relevant.

### 8.3. Rebound Growth and Arrhythmic Risk

Rebound growth after discontinuation has been reported in approximately one-third of cases in one narrative synthesis and in two of the treated pregnancies in the 2026 cohort [21,22]. The phenomenon is biologically plausible because mTOR inhibition suppresses growth signaling without correcting the underlying pathogenic variant. It argues against premature cessation once a response has been achieved and supports serial postnatal echocardiography even when the newborn is initially asymptomatic.

Arrhythmia may persist or appear despite reduction in tumor size. The location of residual tissue at the atrioventricular junction or interventricular septum may be more relevant than total volume. Continuous or repeated electrocardiographic monitoring should therefore form part of neonatal management, particularly after prenatal tachyarrhythmia, pre-excitation or early treatment withdrawal.

## 9. Neurological Outcome and the Limits of Prenatal Disease Modification

**Figure 4:**
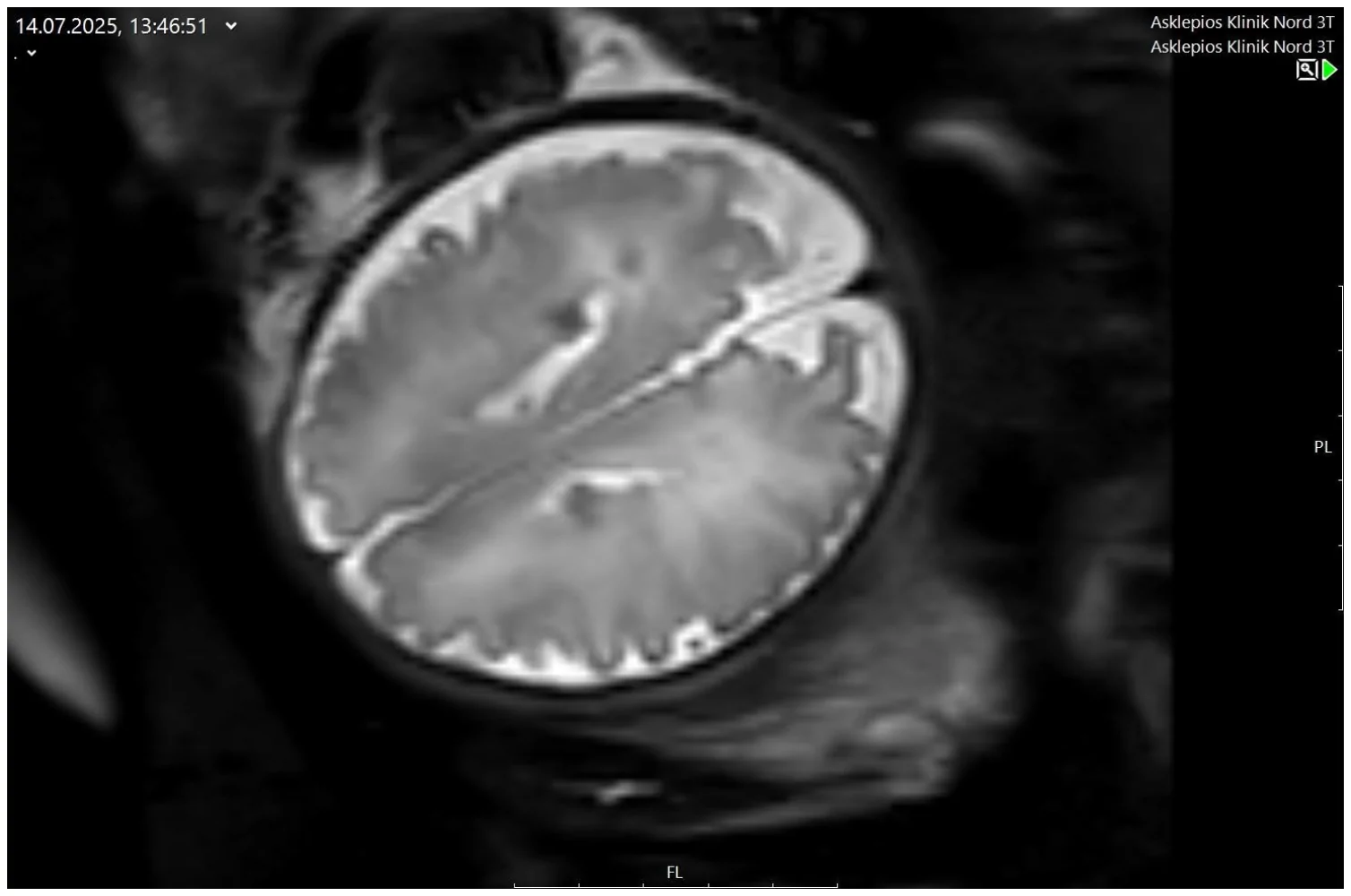
Fetal magnetic resonance imaging demonstrating bilateral T2-hypointense subependymal lesions, compatible with calcified subependymal nodules in the setting of tuberous sclerosis complex.

Cardiac rhabdomyoma is often the first prenatal marker of TSC, and fetal MRI may identify cortical or subependymal lesions (Figure 4). This creates an understandable interest in whether prenatal mTOR inhibition might modify the neurological phenotype. Current evidence is insufficient. Cerebral lesions remained stable in some everolimus-treated cases, but developmental delay, West syndrome and seizures have also occurred despite prenatal exposure [10,12,13]. In the 2026 cohort, short prenatal sirolimus exposure did not produce a consistent reduction in brain-tuber burden [22].

Prenatal treatment should therefore not be offered for presumed neuroprotection outside a research protocol. Cardiological reaction does not automatically match neuroprotection. Postnatal care should follow contemporary TSC recommendations, including early neurological assessment, electroencephalographic surveillance, brain imaging, genetic counseling and systematic evaluation of renal, dermatological and ophthalmological manifestations [3].

## 10. Proposed Clinical Approach

A fetus with a suspected rhabdomyoma should undergo detailed echocardiography, serial assessment of tumor growth and function, targeted neurosonography and counseling regarding fetal MRI and TSC1/TSC2 testing. Low-risk fetuses can be followed at intervals determined by gestational age and growth trajectory. Large, rapidly growing or strategically located tumors warrant assessment at least every 1–2 weeks; weekly review is appropriate once early functional abnormalities appear.

When high-risk physiology is present, multidisciplinary review is required. Counseling should address off-label use, limited evidence, maternal toxicity and rebound. Before treatment, the team should define target trough levels, monitoring, response criteria, the delivery threshold and neonatal transition.

## 11. Limitations of Evidence

The evidence base is small and heterogeneous. Most publications are single-case reports, and treatment was usually offered to fetuses perceived to be at high risk. There is no untreated control with comparable physiology, no standardized response definition and no consensus regarding dose, target level or duration. Spontaneous stabilization, especially later in gestation, complicates attribution of benefit. Conversely, fetuses that deteriorated too quickly to receive treatment may be absent from published literature. Both selection and publication bias are therefore substantial.

Maternal, fetal, and neonatal safety require further evaluation. Long-term fetal safety is also incompletely defined. The rarity of exposure precludes estimation of uncommon adverse effects, and developmental follow-up is short and inconsistent. The reassuring survival reported in early reviews should be interpreted cautiously considering the subsequently published neonatal death and the possibility that arrhythmic risk persists independently of tumor volume [20].

Future studies should use standardized reporting of tumor location and dimensions, obstruction severity, ventricular function, venous Doppler, treatment exposure, maternal trough concentrations, time to response, rebound after cessation, neonatal rhythm and neurodevelopment. An international prospective registry is more realistic than a randomized trial and would provide a denominator for both successful and unsuccessful treatment attempts.

## 12. Conclusions

Prenatal mTOR inhibition can produce rapid regression of fetal cardiac rhabdomyomas and can reverse obstruction, cardiac failure and hydrops. The treatment is most defensible when the fetal problem is progressive: established or impending ventricular inflow/outflow obstruction, deteriorating cardiac function, progressive effusion or hydrops, or clinically significant tumor-associated arrhythmia. Tumor size, multiplicity or a diagnosis of TSC alone should not trigger therapy.

Therapy should be started early enough to permit a response and should generally be continued until sustained stabilization and a coordinated delivery or neonatal treatment plan are in place. Rebound growth and persistent arrhythmic risk make premature discontinuation hazardous. Until prospective registry data are available, transplacental sirolimus or everolimus should remain an individualized rescue or bridging treatment performed in experienced multidisciplinary centers with close maternal monitoring and serial fetal echocardiography.

## 13. What Is Already Known

Fetal cardiac rhabdomyomas are strongly associated with TSC and usually regress spontaneously.

A minority causes obstruction, arrhythmia, heart failure or hydrops and is associated with substantial perinatal risk.

Prenatal maternal sirolimus or everolimus can cross the placenta and reduce tumor size.

## 14. What This Review Adds

The indication for prenatal mTOR inhibition should be based on hemodynamic compromise and documented progression, not on tumor size or TSC diagnosis alone.

Treatment courses of a few days are unlikely to be sufficient; therapy should continue until fetal stabilization, and a safe prenatal-to-postnatal transition is secured.

Rebound growth and persisting arrhythmogenic substrate are clinically important, and favorable tumor regression does not guarantee an uncomplicated neonatal course (Table 1, Table 2, Table 3 and Table 4).

## Figures and Tables

**Table 1:** Proposed physiology-based framework for prenatal mTOR-inhibitor therapy.

Category	Fetal Findings	Clinical Interpretation	Suggested Approach
Strong indication	Established or rapidly progressive LVOT/RVOT obstruction; clinically relevant inflow obstruction; hydrops; deteriorating ventricular function; progressive effusion with venous congestion; tumor-associated arrhythmia with hemodynamic compromise	High risk of fetal decompensation, urgent preterm delivery or critical neonatal obstruction	Discuss transplacental mTOR inhibition promptly in a multidisciplinary fetal therapy setting
Conditional indication	Rapid tumor growth before maturity; tumor approaching a critical valve or outflow tract; increasing AV-valve regurgitation; small but progressive pericardial effusion; recurrent arrhythmia without current hydrops	Risk depends on trajectory, gestational age and feasibility of delivery	Increase surveillance frequency; treat if progression is documented or safe delivery is not yet a reasonable alternative
Insufficient indication in isolation	Tumor size alone; multiple tumors; confirmed TSC; stable non-obstructive lesion; cerebral TSC lesions without cardiac compromise	Likely favorable cardiac natural history; uncertain maternal–fetal risk–benefit ratio	Expectant management with serial fetal echocardiography and TSC-oriented counseling

**Table 2:** Treatment endpoints and practical implications.

Observed Endpoint	Practical Implication
Hydrops resolves and cardiac function stabilizes	Continue close surveillance; maintain treatment if a residual located tumor remains and delivery is not imminent
Inflow/outflow obstruction resolves	Reassess residual anatomy and growth; plan delivery according to fetal cardiac and obstetric status rather than tumor size alone
Fetal maturity is reached	Consider planned delivery in a tertiary center with immediate neonatal echocardiography, rhythm monitoring and an agreed neonatal mTOR-inhibitor strategy
Maternal toxicity develops	Reduce dose or interrupt treatment after multidisciplinary review; increase fetal surveillance and define a delivery threshold
No response after adequate exposure	Confirm adherence and trough level, reconsider diagnosis and mechanism of compromise, and reassess delivery or alternative neonatal management

**Table 3:** Published reports of prenatal mTOR-inhibitor exposure for fetal cardiac rhabdomyoma.

Study	Design/*n*	Primary Fetal Indication	Start/Duration	Agent and Exposure	Prenatal Response	Delivery and Postnatal Outcome	Safety/Interpretative Point
Barnes et al., 2018 [4]	Case report/1	Bilateral outflow obstruction, SVT, impending hydrops	30 weeks to 36 weeks	Sirolimus; target 10–15 ng/mL	Regression after approximately 3 weeks; hemodynamic stabilization	Cesarean at 36 + 3 weeks; rebound after withdrawal; infant sirolimus restarted	No major maternal adverse effect; proof of transplacental exposure
Vachon-Marceau et al., 2019 [5]	Case report/1	Biventricular dysfunction, TR, pericardial effusion	31 + 4 weeks; stopped at 36 weeks	Sirolimus; target 10–15 ng/mL	Marked reduction; improved biventricular function; TR resolved	Delivery at 39 weeks; rebound growth after prenatal cessation	Highlights risk of withdrawal before delivery
Park et al., 2019 [6]	Case report/1	No fetal hemodynamic indication; maternal LAM required therapy	Maternal therapy from 23 weeks to term	Sirolimus	Marked reduction; no mass visible from 29 + 5 weeks	Term delivery; neonatal echocardiography normal	Demonstrates efficacy but not an indication for prophylactic fetal therapy
Pluym et al., 2020 [7]	Case report/1	LVOT obstruction, low output, MR, effusion, impending hydrops	28–36 weeks	Sirolimus; maternal trough 11.6–18.6 ng/mL	Reduced obstruction and MR by 2 weeks; cardiac output normalized by 4 weeks	Vaginal delivery 36 + 6 weeks; stable residual tumors at 6 months	Pre-eclampsia and FGR reported
Ebrahimi-Fakhari et al., 2021 [8]	Case series/3	LVOT obstruction or large tumor with incipient compromise	33–35 weeks; until 36 + 6–39 + 1 weeks	Sirolimus; heterogeneous dosing and trough levels	Regression and relief/prevention of LVOT obstruction	Late-preterm/term delivery; variable developmental follow-up	One fetus with FGR; later speech delay reported in follow-up
Cavalheiro et al., 2021 [10]	Case report/1	Progressive cardiac and cerebral TSC lesions	Late gestation; duration incompletely reported	Everolimus	Cardiac tumor reduced; intracranial lesions stable	Term cesarean; postnatal everolimus; normal neuropsychomotor development at 36 months	Cardiac indication less clearly defined
Dagge et al., 2022 [9]	Case report/1	RVOT obstruction and arrhythmia in fetus of mother with TSC	26 weeks to term	Sirolimus; target 10–15 ng/mL	Tumor regression; rhythm improved but intermittent ectopy persisted	Term cesarean; postnatal sinus rhythm	Maternal therapy had previously been used for renal angiomyolipomas
McLoughlin et al., 2022 [11]	Case report/1	LVOT obstruction, ventricular dysfunction and hydrops	30–36 weeks	Sirolimus; target 10–12 ng/mL	Tumor reduction and improvement of hydrops after approximately 2 weeks	Cesarean at 36 weeks; neonatal inotropes and everolimus; no LVOT obstruction at 3 months	Oligohydramnios prompted delivery
Will et al., 2023 [12]	Case report/1	Right-ventricular inflow obstruction, TR and effusion	27–38 weeks	Sirolimus; trough 8.4–9.9 ng/mL	Reduction and improved ventricular function after 2 weeks	Vaginal delivery 39 + 1 weeks; postnatal everolimus; near-complete regression	Maternal aphthae; developmental delay described during follow-up
Maász et al., 2023 [13]	Case report/1	Progressive cardiac and cerebral lesions	Five-week prenatal course	Everolimus; target 5–15 ng/mL	Tumor reduction	Term delivery; West syndrome at 4 months; mild global delay at 3 years	Shows that cardiac response does not establish neuroprotection
Griesman et al., 2024 [14]	Case report/1	Progressive RVOT obstruction and TR	23–34 weeks	Sirolimus	Tumor decreased after 4 weeks	Vaginal delivery 37 + 6 weeks; recurrent RVOT obstruction at 2 months; sirolimus restarted	Genetic testing negative; maternal aphthae
Schenk et al., 2024 [15]	Case report/1	Progressive intracardiac mass and pericardial effusion	33 + 1 weeks to delivery	Sirolimus; postnatal everolimus	Tumor decreased by birth	Delivery 40 + 4 weeks; normal cardiac and neurological development at 1 year	Limited report; supports planned prenatal-to-postnatal transition
Gonçalves et al., 2025 [16]	Case report/1	Large tumor with ventricular dysfunction and polyhydramnios/effusion	From 29 + 4 weeks to delivery	Sirolimus, dose adjusted to level and toxicity	Progressive reduction and resolution of dysfunction	Cesarean 38 + 2 weeks; no resuscitation; residual non-obstructive tumor	Maternal hypertriglyceridemia improved after dose reduction
Uno et al., 2025 [17]	Case report/1	Severe heart failure, LV inflow/outflow impairment and effusion	33 + 3 weeks to 39 + 2 weeks	Sirolimus; target 5–12 ng/mL	Reduction after 2 weeks; effusion resolved; CVPS improved	Cesarean 39 + 2 weeks; neonatal everolimus; favorable early development	Mild maternal stomatitis
Ritter et al., 2025 [18]	Twin case report/2 exposed; 1 indicated	Giant LVOT-obstructing tumor and hydrops in one twin	28 + 3–34 + 2 weeks	Sirolimus	Hydrops reversed after 4 weeks in affected twin	Both twins delivered; no treatment-related adverse effect reported in minimally affected co-twin	Demonstrates unavoidable co-twin exposure in multiple pregnancy
Bakos et al., 2025 [19]	Single-center series/3 treated	Arrhythmia and/or LVOT obstruction	Individual timing not fully available in aggregate report	mTOR inhibitor	Obstruction resolved in reported treated cases	Aggregate postnatal detail limited	Small retrospective experience; incomplete individual treatment data
Muschel et al., 2025 [20]	Systematic review plus new case/1 new fetus	Imminent bilateral outflow obstruction	23 + 2–28 + 4 weeks; stopped for maternal symptoms	Sirolimus; dose escalated; target 10 ng/mL	Growth arrested by 3 weeks; tumor decreased and obstruction regressed by 5 weeks	Term cesarean; refractory neonatal tachyarrhythmia, capillary leak and death on day 7	Maternal cough prompted cessation; key adverse outcome and arrhythmic warning

**Abbreviations:** CVPS, cardiovascular profile score; FGR, fetal growth restriction; LAM, lymphangioleiomyomatosis; LVOT/RVOT, left/right ventricular outflow tract; MR/TR, mitral/tricuspid regurgitation; SVT, supraventricular tachycardia; TSC, tuberous sclerosis complex.

**Table 4:** Evidence syntheses and cohort data informing clinical interpretation.

Study	Design/Population	Principal Findings	Implication for Practice
Mustafa et al., 2024 [2]	Systematic review and meta-analysis; 400 fetuses overall, including 11 treated with prenatal mTOR inhibition	Treated cases showed reduction in tumor size and improvement in outflow obstruction; no fetal/neonatal death or cardiac surgery among the 11 treated fetuses	Promising comparative signal, but based on uncontrolled reports and susceptible to publication bias
Martinez-Garcia et al., 2025 [21]	Narrative review; 13 studies, 15 treated fetuses	Median treatment start 30 weeks; all reports described regression and cardiac improvement; rebound in 5/15 after cessation	Supports efficacy and identifies rebound as a recurring management problem
Muschel et al., 2025 [20]	Systematic literature review plus new case; 21 exposed fetuses across published experience	Treatment generally initiated at 23–35 weeks; substantial heterogeneity in dose and trough level; new case had favorable prenatal response but neonatal death after early cessation	Provides a broader safety perspective and cautions against equating tumor reduction with elimination of arrhythmic risk
Vergote et al., 2026 [22]	Single-center retrospective cohort; 27 pregnancies, 7 treated with prenatal sirolimus	Untreated growth fastest at 20 + 0–27 + 6 weeks; treatment > 7 days associated with regression and/or resolution of obstruction; rebound after cessation; no consistent effect on brain tubers	Supports sustained rather than very short exposure and a cardiac, not neuroprotective, treatment indication

## Data Availability

No new data were created or analyzed in this study. Data sharing is not applicable to this article.
